# Identification of bacteria and fungi inhabiting fruiting bodies of Burgundy truffle (*Tuber aestivum* Vittad.)

**DOI:** 10.1007/s00203-020-02002-x

**Published:** 2020-07-30

**Authors:** Urszula Perlińska-Lenart, Sebastian Piłsyk, Elżbieta Gryz, Jadwiga Turło, Dorota Hilszczańska, Joanna S. Kruszewska

**Affiliations:** 1 Institute of Biochemistry and Biophysics, Polish Academy of Sciences, Warsaw, Poland; 2 Department of Drug Technology and Pharmaceutical Biotechnology, Medical University of Warsaw, Warsaw, Poland; 3 Department of Forest Ecology, Forest Research Institute, Sękocin Stary, Poland

**Keywords:** *Tuber aestivum*, Bacterial microbiome, Fungal microbiome, Metagenomics analysis, Cultivable microorganisms

## Abstract

*Tuber* species may be regarded as complex microhabitats hosting diverse microorganisms inside their fruiting bodies. Here, we investigated the structure of microbial communities inhabiting the gleba of wild growing (in stands) *T. aestivum*, using Illumina sequencing and culture-based methods. The two methods used in combination allowed to extract more information on complex microbiota of *Tuber aestivum* gleba. Analysis of the V3–V4 region of 16S rDNA identified nine phyla of bacteria present in the gleba of *T. aestivum* ascomata, mostly *Proteobacteria* from the family *Bradyrhizobiaceae*. Our results ideally match the earlier data for other *Tuber* species where the family *Bradyrhizobiaceae* was the most represented. The ITS1 region of fungal rDNA represented six alien fungal species belonging to three phyla. To complement the metagenomic analysis, cultivable fungi and bacteria were obtained from the gleba of the same *T. aestivum* fruiting bodies. The identified fungi mostly belong to the phylum *Basidiomycota* and same to *Ascomycota*. Analysis of cultivable bacteria revealed that all the specimens were colonized by different strains of *Bacillus*. Fungal community inhabiting *T. aestivum* fruiting bodies was never shown before.

**Electronic supplementary material:**

The online version of this article (10.1007/s00203-020-02002-x) contains supplementary material, which is available to authorized users.

## Introduction

Truffles are hypogeous ascomycetous fungi belonging to the genus *Tuber*, which form ectomycorrhizae with trees and shrubs. Some *Tuber* species, produce edible fruiting bodies with a unique flavor and texture and can be regarded as commercial. Two species, *Tuber magnatum* Pico, the white truffle, and *Tuber melanosporum* Vittad., the black truffle, are the most valued by gourmets for their organoleptic properties (Buzzini et al. 2005; Zambonelli et al. 2015). The occurrence of *T. melanosporum* Vittad. is noted mainly from Italy, France, Spain and Balkan countries, such as Croatia and Slovenia and the white truffle (*T. magnatum* Pico) grows exclusively in Italy, Croatia, Slovenia and Hungary (Mello et al. 2006; Büntgen et al. 2011; Pieroni 2016). Increasing attention is drawn toward other *Tuber* species, specifically *Tuber aestivum* Vittad., which exhibits a much wider distribution than any other truffle species. *T. aestivum* has been found in nearly all European countries and beyond, with habitats reaching as far as China and North Africa (Marocco) (Stobbe et al. 2013; Zambonelli et al. 2012). Recent overexploitation and disruption of the natural habitat of the truffle, deforestation, the loss of host plants within forests, the replacement of natural forests with plantations of species that are poor hosts, global warming, acid rain and the loss of expertise during two World Wars as to where and how to harvest mushrooms, particularly truffles (Wang et al. 2013) result in their decreased production. Hence efficient cultivation methods of the valued fungi are highly desirable. In this aspect, a detailed understanding of the interdependences between truffles and their environment is needed. At present, only *T. melanosporum, T. aestivum* and *T. borchii* Vittad. are collected from truffle plantations (Hall et al. 2003; Stobbe et al. 2013; Wang et al. 2013; Zambonelli et al. 2015). In contrast, production of *T. magnatum* in controlled conditions has failed so far (Mello et al. 2017; Zambonelli et al. 2015).

The fruiting bodies of the truffle are hypogeous, it is, therefore, highly likely that soil microorganisms affect their formation (Salerni et al. 2014). During their complex life cycle, truffles establish symbiotic interactions with bacteria (Archaea and Eubacteria) (Antony- Babu et al. 2014; Barbieri et al. 2000, 2005, 2007; Gryndler et al. 2013), fungi (yeasts and filamentous fungi) (Buzzini et al. 2005; Pacioni et al. 2007) and viruses (Stielow and Menzel 2010), at all stages of their development, which include (i) a symbiotic stage in association with the host plant (ectomycorrhiza), (ii) a sexual stage (fruiting bodies) and (iii) a “free living mycelial stage”.

The truffle fruiting body is built of mycelium, in which the outer cells differentiate into a protective layer (peridium) (Pacioni et al. 1995; Zarivi et al. 2015). In *T. aestivum*, the peridium is robust and composed of sclerified and melanized cells with pores forming an outlet of veins which represent authentic entryways (Pacioni 1990). To date, microbes and microbial communities from truffles have been characterized with culture-dependent and independent techniques (Antony-Babu et al. 2014; Barbieri et al. 2000, 2005, 2007, 2016; Benucci and Bonito 2016; Buzzini et al. 2005; Citterio et al. 1995; Gryndler et al. 2013; Nazzaro et al. 2007; Pacioni et al. 2007; Rivera et al. 2010; Sbrana et al. 2002; Splivallo et al. 2015, Stielow and Menzel 2010; Stielow et al. 2011a, b, 2012; Zacchi et al. 2003). Those studies focused mainly on bacteria and rarely on yeast and fungi inhabiting *T. magnatum, T. melanosporum,* and *T. borchii*. Pacioni et al. (2007) also investigated fungi from the fruiting body of *T. aestivum*. Despite those studies, the truffle-inhabiting fungi and bacteria are still poorly characterized, especially those associated with *T. aestivum*. A systematic investigation of fungi living inside the truffle fruiting body has never been performed. Only in recent years, studies on this topic have been initiated in relation to diseases and the shelf life of truffles (Pacioni and Leonardi 2016). Here, we present a microbiome analysis of the gleba of *T. aestivum* fruiting bodies harvested in southern Poland using culture-dependent and culture-independent methods.

## Material and methods

Fruiting bodies of *T. aestivum* were harvested by manual digging from the natural habitats in September 2017, in two localities of Nida Basin (southern Poland). The fruiting bodies signed as 1–3 were sampled in the broadleaved forest with *Quercus petraea* (Matt.) Liebl., *Acer pseudoplatanus* L., *Carpinus betulus* L. at the altitude of 290–296 m a.s.l. and another three fruiting bodies (signed as 4–6) came from the thicket with *Carpinus betulus* L., *Acer campestre* L. and *Populus tremula* L. which grew at 227–228 m a.s.l. The sites were about 50 km apart. Average annual precipitation and temperature in the Nida Basin region (latitude 50°25´N and longitude 20°19´E) are as follow: average annual precipitation is 600 mm, and average temp. 8.0 °C. Soil conditions of the *T. aestivum* growing sites were characterized previously by Hilszczańska et al. (2008, 2014).

**Fig. 1:**
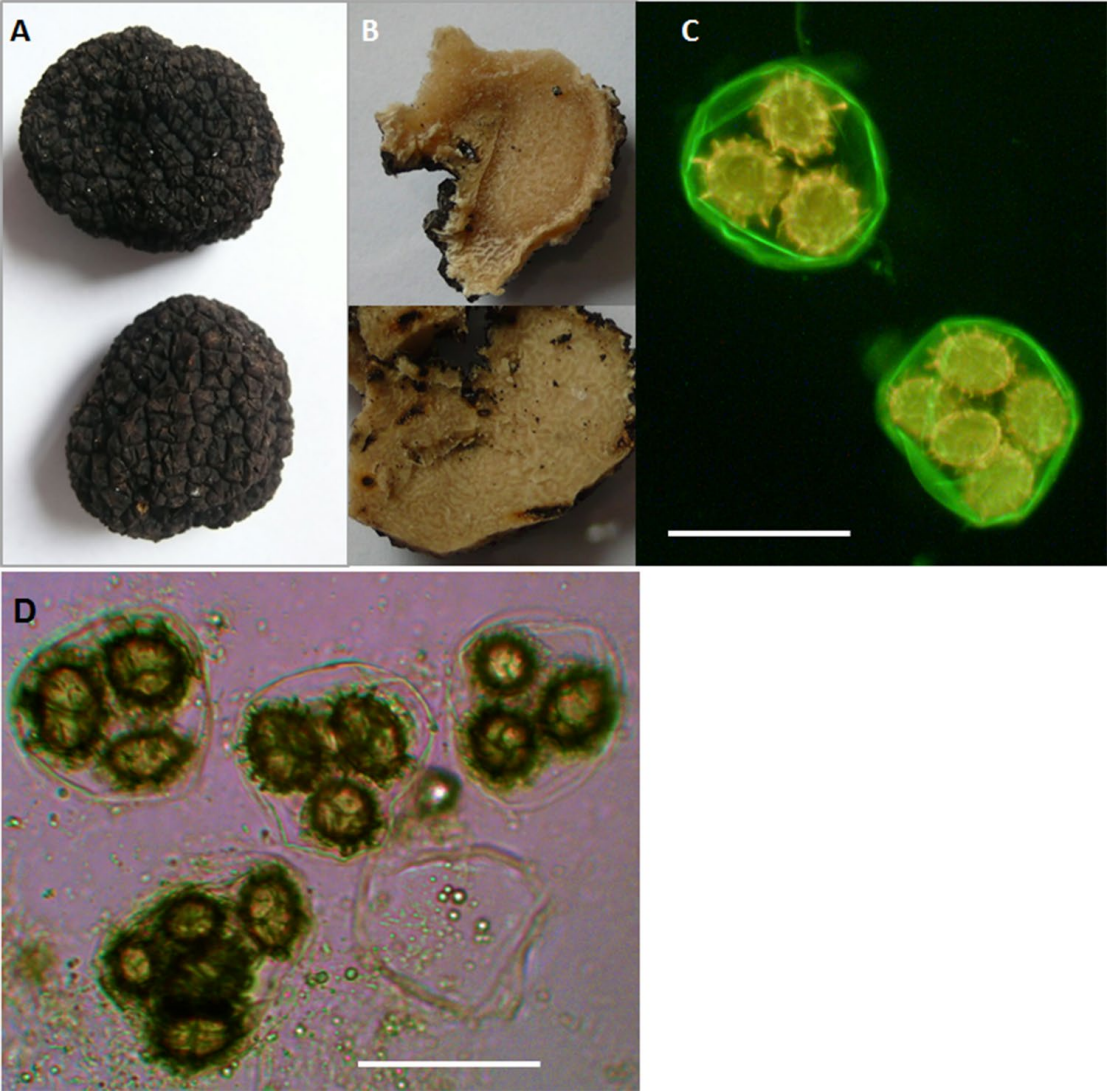
*Tuber aestivum* ascomata **a**, **b** and asci with ascospores **c**, **d**. A—*T*. *aestivum* fruiting bodies. **b**—fruiting bodies cut for gleba isolation. **c**—asci with ascospores stained with Atto488 dye conjugated with wheat germ lectin (Sigma-Aldrich) which binds to chitin in asci and ascospores. **d**—Evaluation of spores melanization in the studied specimens. Samples were examined using a Delta Optical microscope at 40 × magnification. Bar = 50 µm

The harvested truffles were unwashed, separately packed into vacuum boxes and immediately transferred to the laboratory for analysis (Fig. 1). The species identity was determined by DNA analysis (Martin and Rygiewicz, 2005; Weden et al. 2005). DNA was extracted from gleba of *T. aestivum* using Plant DNA Mini Kit (Syngen Biotech, Poland). Extracted DNA was used as the template for amplification of the ITS region using universal primers ITS1 and ITS4 (White et al. 1990) and a standard PCR protocol (Martin and Rygiewicz 2005). Amplified products were separated by electrophoresis on an agarose gel, isolated from the gel by Qiaex II Gel extraction kit (Qiagen) and sequenced in the Institute of Biochemistry and Biophysics PAS using ABI3730XL DNA Analyzer (Thermo Fisher Scientific Inc. Waltham, MA, USA).

The obtained sequences were compared to the sequences of *Tuber aestivum* isolated in Poland KX028767, KX028766 (Hilszczańska et al. 2016) and deposited in the Genbank database NCBI (Benson et al. 2013). For analysis only, fully mature ascocarps with 71–100% asci containing melanized spores (Büntgen et al. 2017) were chosen. Melanization was evaluated under a Delta Optical microscope (Fig. 1d).

### Preparation of 16S rDNA and ITS1 amplicon libraries, DNA sequencing and data analysis

Six *T. aestivum* ascocarps were subjected to metagenomic analysis.

For surface sterilization, they were immersed in 70% ethanol for 2 min. following by 5 min. in 5% sodium hypochlorite and then rinsed three times (1 min. each) with sterile distilled water. After drying on sterile filter paper, the fruiting bodies were cut and 100 mg samples of glebal tissue homogenized in liquid nitrogen were taken for DNA isolation using Plant DNA Mini Kit (Syngen Biotech, Poland), following the manufacturer’s protocol. Quality of the DNA was checked on the basis of electrophoregram in 1% TAE-agarose gel. Isolated DNA was stored at  – 20 °C. The gleba was also collected for the cultivation of fungi and bacteria for further analysis.

Bacterial and fungal taxa were identified based on 16S rRNA gene sequence and the ITS1 rDNA region, respectively (Haas et al. 2011; Kõljalg et al. 2013; Martin and Rygiewicz, 2005; Thijs et al. 2017). Since no representatives of Archaebacteria were identified in the present study, we use the term “bacteria “ to represent Prokaryote throughout the text. The V3–V4 variable region of 16S rDNA was amplified in the total volume of 25 µl using 5 µl of 1 µM primers 341F and 785R (Kõljalg et al. 2013) and the ITS1 region using primers ITS1FI2 (Schmidt et al. 2013) and 5.8S (Vilgalys and Hester 1990) (Table 1). PCR reactions were performed using 2.5 µl (5 ng/µl) of DNA template and 12.5 µl of 2xKAPA HiFi Hot Start Ready Mix (Kapa Biosystems) and following PCR. The same standard conditions were used for both ITS and 16S: initial denaturation at 95 °C for 3 min, followed by 25 cycles of 95 °C for 30 s, 55 °C for 30 s, 72 °C for 30 s with a final extension step at 72 °C for 5 min (White et al. 1990; Thijs et al. 2017). Sequencing of the PCR products was done by Genomed (Warsaw, Poland) using Illumina MiSeq Instrument and pair-end (2 × 250 bp) mode with V2Illumina kit (Balint et al. 2014). Control reaction was performed without DNA added. A preliminary sequence analysis was performed using MiSeq Reporter (MSR) v2.6 program and full comparative analysis using QIIME (Caporaso et al. 2010) with the GreenGenes v13-8 database as a reference for bacteria and UNITE v7 reference database for fungi. Singletons (OTUs represented by a single sequence) were excluded from data analysis (minimal OTU count = 10) and OTUs were picked with sequence identity criteria of 97%.

**Table 1:** Primers used for amplification of rDNA fragments

Primer name	5′- Sequence -3’	Purpose	References
341F	CCTACGGGNGGCWGCAG	Bacterial 16S rDNA amplification in metagenomic study	Thijs et al. 2017
785R	GACTACHVGGGTATCTAATCC		
ITS1FI2	GAACCWGCGGARGGATCA	Fungal specific ITS1 amplification in metagenomic study	Schmidt et al. 2013
5.8S	CGCTGCGTTCTTCATCG		Vilgalys and Hester 1990
F27	AGAGTTTGATCMTGGCTCAG	Identification of cultured bacteria	Frank et al. 2008
R1492	TACGGYTACCTTGTTACGACTT		
ITS1	TCCGTAGGTGAACCTGCGG	Identification of cultured fungi	White et al. 1990
ITS4	TCCTCCGCTTATTGATATGC		

### Cultivation of fungi and bacteria from gleba of *T. aestivum* fruiting bodies

To cultivate fungi inhabiting gleba of the truffles, gleba samples collected as described above were transferred to Petri dishes containing PDA medium (Potato Dextrose Agar) supplemented with chloramphenicol (0.01%), to inhibit bacterial growth and Bengal rose (0.005%), to inhibit overgrowth by rapidly growing molds and to facilitate isolation of slow-growing fungi (King et al. 1979) and cultivated at 28 °C. Single fungal colonies were sub-cultured on separate Petri dishes containing PDA medium to obtain pure isolates for further studies. Pure culture isolates have been deposited at the culture collection of the Laboratory of Fungal Glycobiology IBB PAS, Warsaw, Poland.

For DNA extraction, mycelia were cultivated in PDB medium (Potato Dextrose Broth) at 28 °C on a rotary shaker (250 rpm) in 250 ml shake flasks containing 100 ml of medium. DNA was isolated using the Wizard Genomic DNA Purification kit (Promega, Mannheim, Germany). Extracted DNA was used as the template for amplification of the ITS region using universal primers ITS1 and ITS4 (White et al. 1990) and a standard PCR protocol (Martin and Rygiewicz 2005). Amplified products were separated by electrophoresis on an agarose gel, isolated from the gel by Qiaex II Gel extraction kit (Qiagen) and sequenced. DNA sequences were analyzed using the NCBI database and the BLAST algorithm (Altschul et al.^1997^).

Identified species were classified according to the MycoBank fungal database (nomenclature and species bank) https://www.mycobank.org.

For isolation of cultivable bacteria, gleba samples were homogenized in sterile 0.85% NaCl and plated on Tryptone Soy Agar (TSA) (Sigma–Aldrich) as described (Barbieri et al. 2005). Single colonies were sub-cultured on separate Petri dishes containing TSA medium to obtain pure isolates.

DNA was extracted from bacterial colonies (220 pure isolates)) resuspended in TES buffer (20 mM Tris, 50 mM EDTA, 150 mM NaCl, pH 7.9) and lysed with lysozyme (5 mg ml^−1^) (Sigma –Aldrich) and incubated at 37 °C for 1 h. DNA was extracted lysed with lysozyme using standard phenol extraction as described by Barbieri et al. (2005). Isolated DNA was used as the template for amplifying the DNA between positions 27 and 1492 of bacterial 16S rRNA genes (numbered according to the *Escherichia coli* rRNA) using primers F27 and R1492 and standard PCR protocol (Frank et al. 2008). DNA sequences were analyzed as above (Altschul et al. 1997). Acc. no. for bacterial strains deposited in NCBI are shown in the Table 5.

### Results

### Bacterial community inhabiting gleba of *T. aestivum*

The microbiome of the gleba was determined individually for six truffle fruiting bodies. Illumina sequencing detected 1,012,979 classified bacterial reads for six fruiting bodies (Table 2). 16% of the classified reads (163,937 reads) has been assigned to 20 bacterial OTUs (Table 1S-6S) that belonged to 9 bacterial phyla, 13 classes, 14 orders and 22 families.

**Table 2:** Assignment of sequencing reads from six fruiting bodies of *T. aestivum* to bacterial phyla

16S rDNA V3–V4 region	Specimen no					
	1	2	3	4	5	6
No. of total reads	187,597	183,746	174,153	180,784	204,455	187,333
No. of classified reads	169,765	168,092	159,646	160,900	186,576	168,000
Proteobacteria	158,027	162,793	153,670	143,731	180,198	148,653
Bacteroidetes	5895	894	1141	4300	549	17,019
Actinobacteria	782	2775	3017	7875	3471	1822
Chloroflexi	537	165	278	837	456	149
Verrucomicrobia	414	165	232	1094		
Acidobacteria		277		1914	690	61
Firmicutes	155	652			712	185
Planctomycetes				415		60
TM7			475			

Illumina sequencing of variable region V3–V4 of 16S rDNA amplified from total gleba DNA was used to identify bacterial phyla

Analysis showed that *Proteobacteria* dominated the bacterial community (Table 2 and Table 1S-6S), with 88.48–96.80% of all identified sequences belonging to the phylum *Proteobacteria*. The second most common phylum was *Bacterioidetes*: 2.67–10.13% sequences were assigned to this phylum in three fruiting bodies (1, 4 and 6). Six more phyla were only modestly represented: *Acidobacteria, Chloroflexi, Firmicutes, Planctomycetes, Verrucomicrobia,* and *TM7* a candidate phylum a close relative of the *Chloroflexi*.

**Fig. 2:**
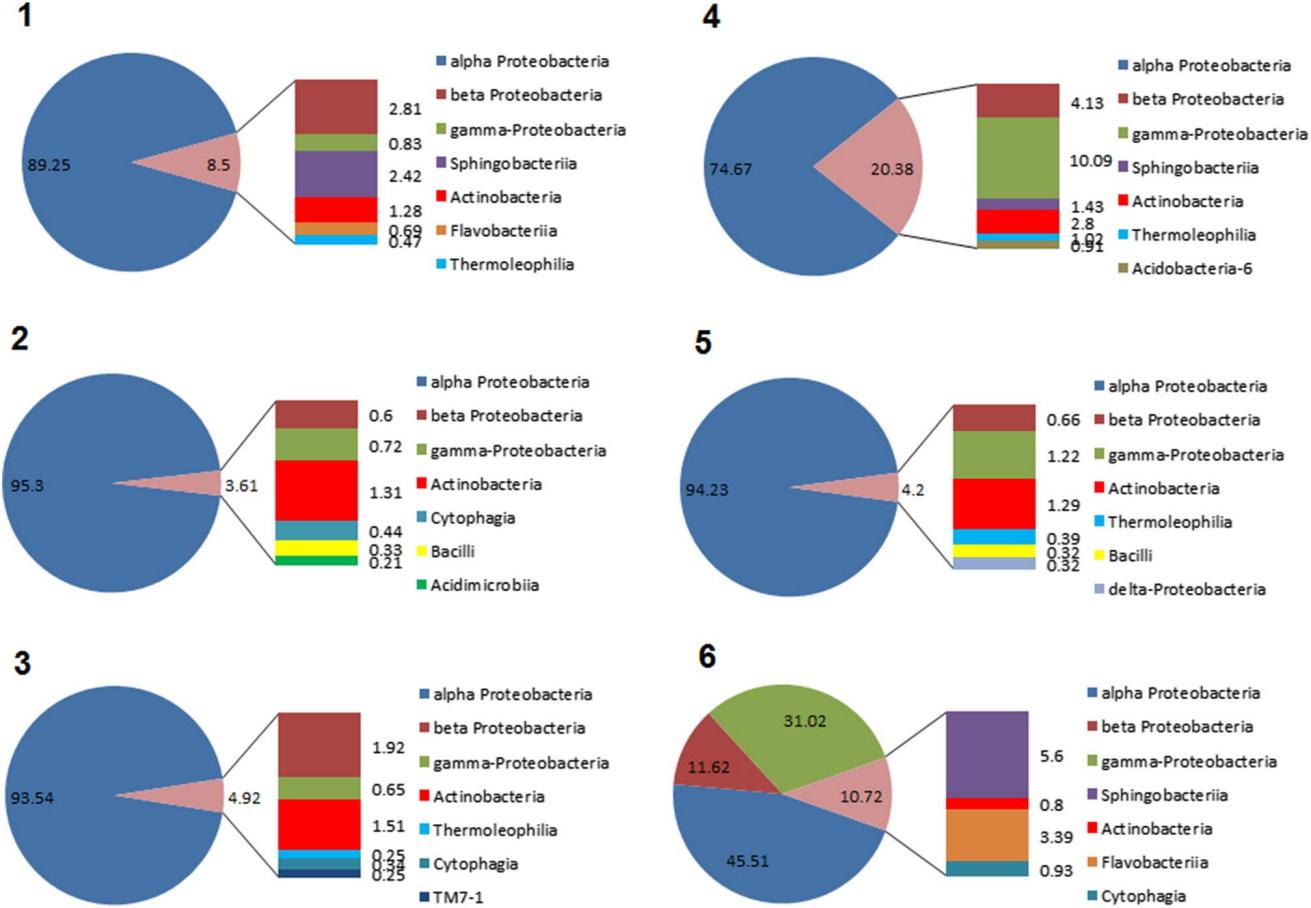
Bacterial diversity at class level in gleba of six fruiting bodies of *T. aestivum*. Illumina sequencing of variable region V3-V4 of 16S rDNA amplified from total gleba DNA was used to identify bacterial classes. (For details see Table 1S-6S). Percentage of qualified reads assigned to each bacterial class is shown

The microbiome of specimen 6 was different from the others, which was well visible at the class level (Fig. 2 and Tables 1S–6S). In this sample, only 45.51% of the sequences were assigned to alpha-*Proteobacteria* while in the other fruiting bodies this proportion varied from 74.67 to 95.3%. In specimen 6, 31.02% of the sequences belonged to gamma-*Proteobacteria*, which were represented by a significant fraction of sequences (10.09%) in only one other specimen, no.4 (Fig. 2 and Tables 1S–6S).

Class alpha-*Proteobacteria* was represented by five families mostly from the order *Rhizobiales.* Family *Bradyrhizobiaceae* from this order was the most common in specimen 2 where 94.31% of sequences were assigned to this family, while in specimen 6 only 41.11% (Tables 1S–6S). In the specimen, 6 family *Pseudomonadaceae* belonging to gamma-*Proteobacteria* was more common (30.61%) than in any other specimens, where the percentage of sequences assigned to *Pseudomonadaceae* varied from 0 to 8.73%.

In total, 20 strains were identified to the genus level. Genus *Acidivorax* belonging to beta-*Proteobacteria*, order *Burkholderiales*, family *Comamonadaceae* was identified in all six specimens. Family *Comamonadaceae* was also represented by genera *Variovorax*, found in four specimens, and *Roseateles* found only in specimen 4. Eight genera of bacteria were found only in a single specimen (Tables 1S–6S).

### Fungal community in *T. aestivum* gleba

To characterize the fungal microbiome associated with the gleba of *T. aestivum* fruiting bodies we analyzed the ITS1 (internal transcribed spacer) region of rDNA in total DNA individually for six truffle specimens.

A total of 599,375 classified reads were obtained from the six specimens (Table 3), a vast majority (from 99.74 to 99.99%) was assigned to *T. aestivum* OTU. However, sequences assigned to other fungal OTUs from three phyla (0.03% *Ascomycota* other than *Tuber*, 0.03% *Basidiomycota* and 0.006% *Mucoromycota*) were also detected.

**Table 3:** Assignment of sequencing reads from six fruiting bodies of *T. aestivum* to fungal phyla

ITS	Specimen no					
	1	2	3	4	5	6
No. of total reads	144,342	126,150	128,755	132,889	111,526	132,390
No. of classified reads	116,000	96,763	99,876	104,187	84,883	97,666
*T. aestivum*	115,701	96,745	99,867	104,111	84,866	97,593
Ascomycota (excluding *T. aestivum*)	88	12		70	4	7
Basidiomycota	166	1		1	6	3
Mucoromycota	29	1		5		

Illumina sequencing of ITS1 region of fungal rDNA

**Fig. 3:**
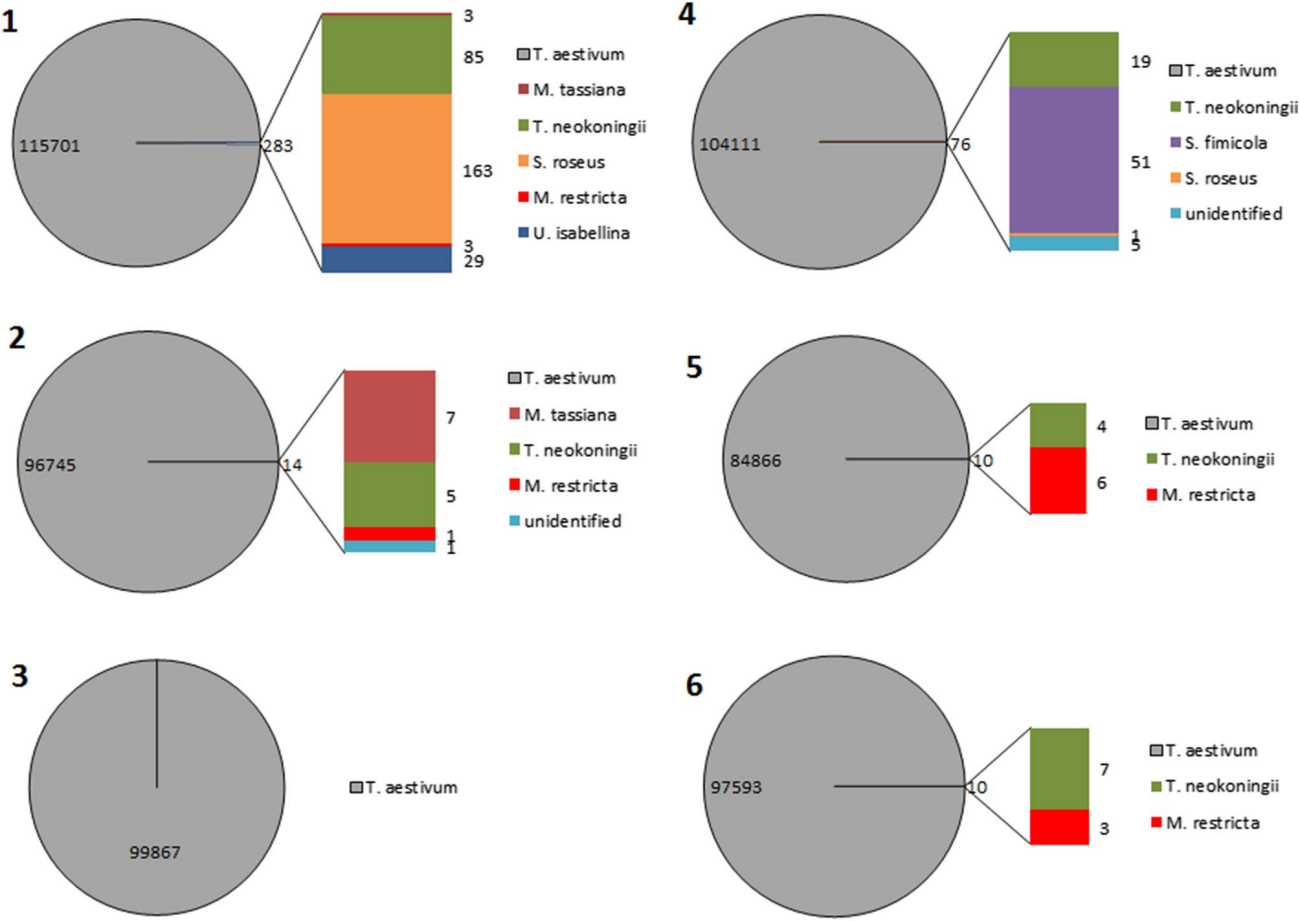
Fungal diversity in gleba of six fruiting bodies of *T. aestivum*. Illumina sequencing of ITS1 region of fungal rDNA amplified from total gleba DNA was used to identify fungal species. Number of qualified reads assigned to the identified species is shown

In all, six fungal species were found to reside inside the examined fruiting bodies of *T. aestivum* (Fig. 3 and Table 7S).

The largest variety of fungal sequences was found in specimen 1(five additional species) and in specimens 2 and 4 (three species each). In specimens 5 and 6 two fungal species were identified. No alien fungi were identified in specimen 3.

Sequences representing *Trichoderma neokoningi* Samuels & Soberanisan and *Malassezia restricta* E. Guého, J. Guillot & Midgley were found in five (specimens 1, 2, 4, 5, 6) and four (specimens 1, 2, 5, 6) fruiting bodies, respectively, and those representing *Mycosphaerella tassiana* (De Not.) Johanson, in two specimens (specimen 1 and 2) and *Umbelopsis isabellina* (Oudem.) W. Gams (specimen 2) and *Sphaerodes fimicola* (E.C. Hansen) P.F. Cannon & D. Hawksw (specimen 4) – in a single specimen only.

### Cultivable fungi and bacteria inhabiting *T. aestivum* gleba

For a complete picture of the fungal community inhabiting the fruiting bodies of *T. aestivum* the alien fungi were grown directly from the gleba samples. Ten cultivable fungal species were identified with identity from 83 to 99% (Table 4) and an additional strain could be identified only to the family level. The obtained sequence of *Phlebia* (Table 4) was in 99% identical to *P. rufa* and *P. radiate*, as well.

**Table 4:**
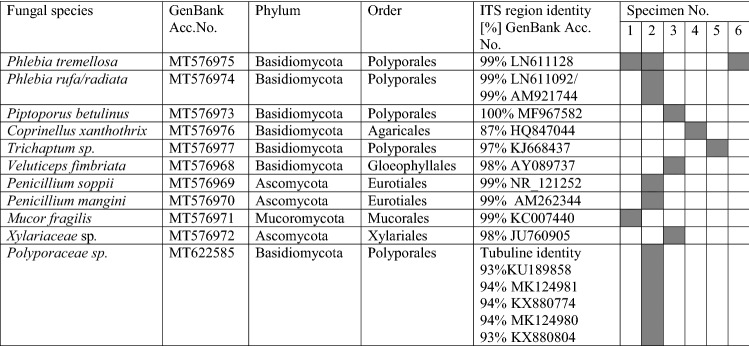
Cultivable fungi isolated from gleba of *T. aestivum* fruiting bodies

Grey color indicates presence of the identified fungus in the specimen

Most of the identified fungi belonged to the phylum *Basidiomycota*, four and two representing the *Polyporales* and *Agaricales* orders, respectively, and one belonged to order *Gloeophyllales*. The other four species were assigned to the phyla *Ascomycota* and *Mucoromycota*. Notably, none of these isolates corresponded to the fungal species identified above by metagenomics sequencing.

The *T. aestivum* gleba was also analyzed in a similar manner for cultivable bacteria. We used Tryptone Soy Agar (TSA) medium which has been used earlier to study cultivable bacteria from the ascocarps of *T. borchii* and *T. magnatum* (Barbieri et al. 2000, 2005, 2007). Cultivable bacteria were identified with identity at least 96% (Table 5), one obtained sequence was identical in 97% with *Bacillus simplex* and *Bacillus huizhouensis* 16S sequence. The same level of identity was obtained for the second sequence identified as *B. toyoensis* and *B. thuringensis* 16S sequences.

**Table 5:**
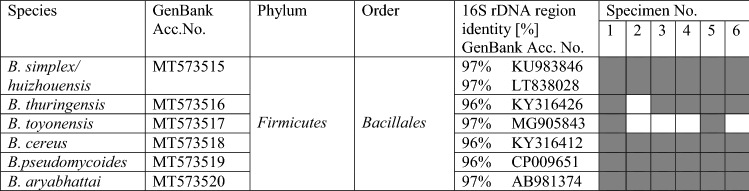
Cultivable bacteria isolated from gleba of *T. aestivum* fruiting bodies

Grey color indicates presence of the identified bacteria in the specimen

**Fig. 4:**
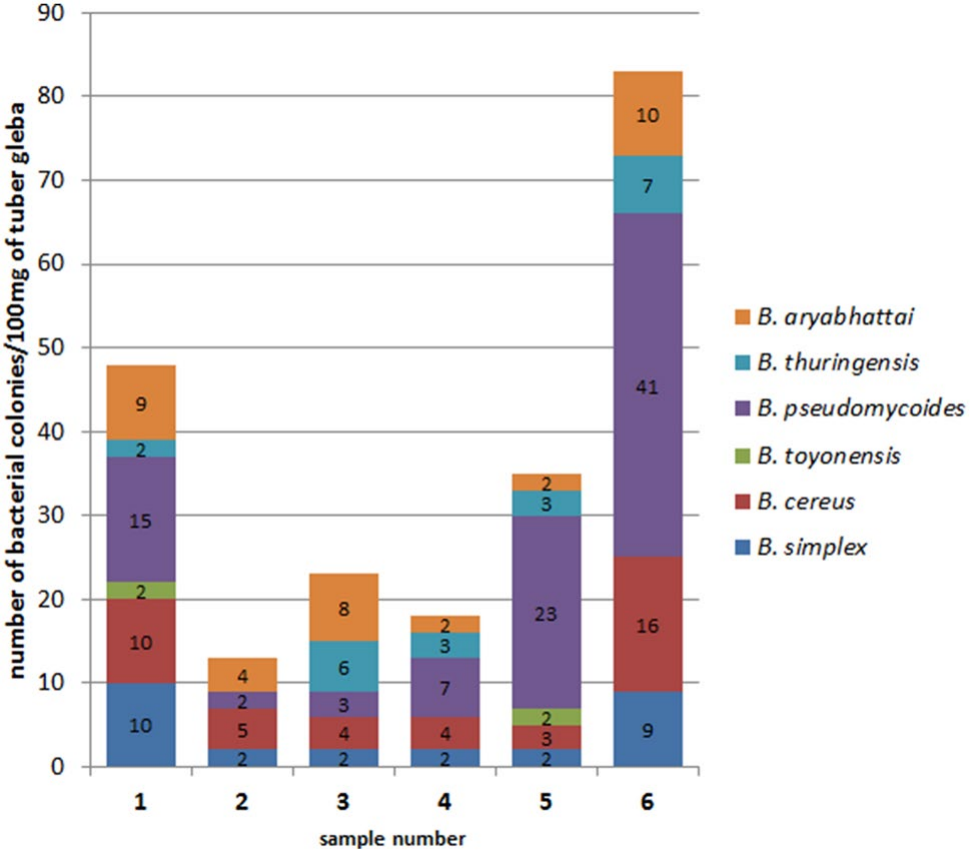
Abundance and diversity of cultivable bacteria in gleba of six fruiting bodies of *T. aestivum*. Number of colonies of *Bacillus* species indicated obtained from 100 mg of gleba is shown

All the specimens were colonized by different strains of genus *Bacillus*, family *Bacillaceae*, order *Bacillales,* class *Bacilli*, phylum *Firmicutes* (Table 5 and Fig. 4). The largest number of bacterial colonies were grown from specimen 6 and the lowest from specimen 2. *Bacillaceae* family was not assigned to any of the samples in the metagenomics studies.

These results were fundamentally different from those described above for the metagenomics approach. There, order *Bacillales* was only represented by *Staphylococcus aureus*, family *Staphylococcaceae* (in specimens 1, 2 and 5) and no representative of family *Bacillaceae* was identified.

## Discussion

### Bacterial microbiome

Truffles host a diverse microbiome inside their fruiting bodies, comprising bacteria (Antony-Babu et al. 2014; Barbieri et al. 2005, 2007, 2016; Benucci and Bonito 2016; Citterio et al. 1995; Gryndler et al. 2013; Pacioni 1990; Sbrana et al. 2002; Splivallo et al. 2015), yeasts (Buzzini et al. 2005) filamentous fungi (Pacioni et al. 2007) and viruses (Stielow and Menzel 2010; Stielow et al. 2011a, b, 2012).

The first study of the bacterial microbiome of *T. borchii* revealed that most 16S rDNA sequences belonged to alpha-*Proteobacteria*, with over 97% of them assigned to *Rhizobium* and *Bradyrhizobium* spp. (Barbieri et al. 2005).

The factors which regulate microorganisms’ association with the truffle gleba are unknown. Antony-Babu et al. (2014) hypothesized that particular bacterial communities could participate in the truffle development and are selected to the microbiome of the ascocarp during its maturation. A detailed study by that group has shown that the gleba microbiome of *T. melanosporum* evolves during ascocarp formation. Differences in the microbiome composition have also been shown for the maturing ascocarp of *T. magnatum*, where alpha-*Proteobacteria* mainly from the genera *Sinorhizobium*, *Rhizobium* and *Bradyrhizobium* were identified (Barbieri et al. 2007).

The bacterial communities of several *Tuber* species (*T. oregonense* Trappe, Bonito & Rawlinson*, T. gibbosum* Harkn.*, T. lyonii* Butters*, T. melanosporum, T. indicum* Cooke & Massee) from different geographical regions (Europe, USA and Asia) revealed significant differences in their OTUs but in all specimens sequences from *Bradyrhizobium* spp. alpha-*Proteobacteria* were dominant (Benucci and Bonito 2016).

Results of this study, concerning *T. aestivum* ideally match the earlier data for other *Tuber* species. We found out that the bacteria of family *Bradyrhizobiaceae* were the most abundant inhabitants in all examined specimens of *T. aestivum*. It seems the bacteria belonging to this family also dominate in case of all truffle species. Additionally, the fruiting bodies of *T. aestivum* (this study), *T. borchii* (Barbieri et al. 2005) and *T. magnatum* (Barbieri et al. 2007) all contained such bacteria as *Bosea* spp. (*Bradyrhizobiaceae*), but also beta-*Proteobacteria,* (*Variovorax* spp.) and gamma-*Proteobacteria,* (*Pseudomonas* spp.). *Pseudomonas* has also been isolated by Citterio et al. (1995) from the fruiting bodies of *T. magnatum*, *T. borchii* and *T. maculatum* Vittad. together with *Staphylococcus* belonging to the phylum *Firmicutes* (class *Bacilli*). We also found *Staphylococcus* spp. in three specimens of *T. aestivum*. On the other hand, *Staphylococcus* spp. seems to be widely represented in the microbiome of fungal fruiting bodies since it was found also in fruiting bodies of forest mushrooms *Agaricales, Boletales, Russulales* and *Cantharellales* all from *Basidiomycota* phylum (Pent et al. 2017).

The overall similarity of the bacterial communities inhabiting different *Tuber* species suggests that, regardless of the species, the truffle fruiting body creates a specific habitat for a core bacterial microbiome (Splivallo et al. 2015).

The core bacterial microbiome is supplemented specifically depending on the truffle species and the environment. Thus, *Acidovorax* spp. (beta-*Proteobacteria*) was identified in all specimens of *T. aestivum* (this study), but not in *T. borchii* or *T. magnatum* ascocarps (Barbieri et al. 2005, 2007). Furthermore, to the best of our knowledge, another beta*-Proteobacteria*, *Cupriavidus* sp., found in four of our specimens of *T. aestivum* has not been detected so far in any of the truffle species studied.

Our cultivation-based approach identified six strains of *Bacillus* not represented in the molecular analysis. However, there were general similarities between the two approaches, both of which found the highest bacterial diversity in specimen 6 and the lowest in specimen 2.

On the other hand, Barbieri et al. (2005) noticed that many species of bacteria resist cultivation because of their interdependencies with other microbes or because of the lack of knowledge concerning their specific growth requirements. Given that a large portion of environmental bacteria has not been cultured yet, the bacteria isolated thus far could represent only a fraction of the entire natural bacterial community associated with truffles.

### Fungal microbiome

The truffle fruiting bodies have been shown to host not only bacteria but also yeasts and filamentous fungi (Buzzini et al. 2005; Pacioni et al. 2007).

Pacioni et al. (2007) analyzed guest cultivable fungi from the gleba of ten *Tuber* spp. They failed to obtain any mycelial isolates from *T. aestivum* despite having studied nine specimens but did isolate several filamentous fungi from other *Tuber* species. In this study, we identified eleven cultivable fungi from the gleba of *T. aestivum*. Four strains were assigned to the order *Polyporales* not represented in any other *Tuber* species described by Pacioni et al. (2007). In contrast, the culture-independent molecular studies of the fungal microbiome of *T. aestivum* gleba identified *Trichoderma neokoningi* from the order *Hypocreales* and this order was represented in other *Tuber* species although not by *Trichoderma* (Pacioni et al. 2007).

Identified fungi *T. neokoningi*, *Mycosphaerella tassiana* and *Umbelopsis isabellina* are common soil and plant root inhabiting species while *Sphaerodes fimicola* was reported to be a coprophilous fungus found in deer fecal samples (Caretta and Piontelli 1996). It is known that truffle spores are passively dispersed by animals (Laessoe and Hansen 2007; Ori et al. 2018). Together with truffles, they could also potentially distribute other fungi as *S. fimicola* or *Malassezia restricta*. However, we do not know if the soil fungi play a role in the development of *T. aestivum* fruiting body or they are just present in the soil because of other reason such as the spread of feces.

The data on the fungal microbiome of *Tuber* spp. fruiting bodies is limited but more information regarding the fungi inhabiting the soil and root systems of plants infected by truffle are available (Benucci et al. 2011; Leonardi et al. 2013; Li et al. 2017; Mello et al. 2010, 2011; Napoli et al. 2010; Pruett et al. 2008; Zacchi et al. 2003). As could be expected, some such fungi are typical mycorrhizal microorganisms (Benucci et al. 2011; Leonardi et al. 2013; Li et al. 2017) characteristic for each tree and its surrounding (Beckers et al. 2017; Murat et al. 2005). The two localities where truffles were harvested differed in the type of stand. Specimens 1–3 were sampled in the broadleaved forest with *Quercus petraea*, *Acer pseudoplatanus*, and *Carpinus betulus* and specimens 4–6 come from the thicket with *Carpinus betulus*, *Acer campestre* and *Populus tremula*. In addition, the microbial community in the vicinity of *Tuber* ectomycorrhize could be modulated by the presence of the *Tuber* mycelia (Leonardi et al. 2013). Those authors observed that the dominance of *Tuber* mycelia resulted in a lower diversity and abundance of endophytic pathogenic fungi and demonstrated that the bacterial diversity of the ectomycorhizosphere soil was significantly lower than that of more distant soil. This phenomenon could be a plausible explanation of why the community of bacteria and fungi in the *Tuber* gleba is even more limited. However, since we did not investigate microorganisms inhabiting soil at our location, it is only our supposition.

Another significant phenomenon is that the *Tuber* fruiting bodies selectively recruit microorganisms to their gleba, but the criteria and mechanisms of this recruitment remain unknown. Notably, we found that the microbiomes of individual fruiting bodies differed from one another markedly, although all the specimens were at the same stage of maturation.

The role of the bacterial and fungal communities residing inside fruiting bodies is controversial but it has been proposed that the bacteria could participate in the development and maturation of truffles (Antony-Babu et al. 2014; Barbieri et al. 2010; Buzzini et al. 2005; Splivallo et al. 2015; Splivallo and Ebeler 2015; Vahdatzadeh et al. 2015—review). It is also assumed that they contribute to the characteristic flavor, which may vary quite substantially between specimens (Vahdatzadeh et al. 2015 – review).

The same role could be attributed to fungi. *Sporobolomyces roseus* found in our samples of *T. aestivum* gleba and previously reported as a grape-associated fungus could produce aromatic compounds typical for the red wine aroma (Verginer et al. 2010). Fungi could also play a protective role in producing antibacterial metabolites, which has been shown for *Trichopezizella nidulus* (J.C. Schmidt & Kunze) Raitv. found in *Tuber nitidum* Vittad. and *Talaromyces wortmannii* (Klöcker) C.R. Benj. found in *T. rufum* Picco (Bara et al. 2003; Pacioni et al. 2007; Thines et al. 1998).

In summary, our study has shown that individual fruiting bodies of *T. aestivum* collected from two areas, representing the same stage of maturity, possessed different microbiomes. These differences may result from the diverse plant community of forest floor environment (Hilszczańska et al. 2018) and, therefore, influence differences in microbiomes. In general, the core bacterial microbiome of the studied gleba from *T. aestivum* was similar to the bacterial communities of the other *Tuber* species (Benucci and Bonito 2016).

Most studies focused on bacterial communities and their role in truffle species. Knowledge about the fungal microbiome and its functions in Tuber spp. is very limited. The fungal microbiome should be examined in detail, and the test should be carried out not only for T. aestivum but also for other *Tuber* spp.

## Electronic supplementary material

Below is the link to the electronic supplementary material.

Supplementary file1 (DOCX 31 kb)

Supplementary file2 (DOCX 15 kb)
