# Supplementary material for: Identification of bacteria and fungi inhabiting fruiting bodies of Burgundy truffle (Tuber aestivum Vittad.)
Source: Arch Microbiol. 2020 Jul 30;202(10):2727–38. doi: 10.1007/s00203-020-02002-x (PMC7538415; doi:10.1007/s00203-020-02002-x)
Supplement: Supplementary file 1 — Supplementary file1 (DOCX 31 kb) [file 203_2020_2002_MOESM1_ESM.docx]

Table 1S

Total classified reads obtained for specimen 1 assigned to bacterial taxons

| Phylum | Class | Order | Family | Genus | Species |
| --- | --- | --- | --- | --- | --- |
| Proteobacteria 158027 | alpha-Proteobacteria 151565 | Rhizobiales 150817 | Bradyrhizobiaceae 102165 |  |  |
|  |  |  | Phyllobacteriaceae 46013 | Phyllobacterium 45965 |  |
|  |  |  | Rhizobiaceae 1.00 |  |  |
|  | beta-Proteobacteria 4746 | Burkholderiales 4679 | Comamonadaceae 4436 | Variovorax 2277 | V. paradoxus 2269 |
|  |  |  |  | Acidovorax 1227 |  |
|  |  |  | Burkholderiaceae 78 | Cupriavidus 78 |  |
|  | gamma-Proteobacteria 1413 | Xanthomonadales 716 |  |  |  |
| Bacteroidetes 5895 | Sphingobacteria 4113 | Sphingobacteriales 4113 | Sphingobacteriaceae 3845 | Pedobacter 3845 |  |
|  | Flavobacteria 1166 | Flavobacteriales 1166 | Flavobacteriaceae 1166 | Flavobacterium 1166 |  |
| Actinobacteria 3627 | Actinobacteria 2178 | Actinomycetales 2125 | Mycobacteriaceae 549 | Mycobacterium 549 |  |
|  | Thermoleophilia 802 | Gaiellales 594 |  |  |  |
| Acidobacteria 782 |  |  |  |  |  |
| Chloroflexi 537 |  |  |  |  |  |
| Verrucomicrobia 414 |  |  |  |  |  |
| Firmicutes 155 | Bacilli | Bacillales | Staphylococcaceae | Staphylococcus | S. aureus 77 |

minimal OTU count = 10

Table 2S

Total classified reads obtained for specimen 2 assigned to bacterial taxons

| Phylum | Class | Order | Family | Genus | Species |
| --- | --- | --- | --- | --- | --- |
| Proteobacteria 162793 | alpha-Proteobacteria 160277 | Rhizobiales 159571 | Bradyrhizobiaceae 158630 |  |  |
|  |  |  | Hyphomicrobiaceae 534 | Rhodoplanes 361 |  |
|  | beta-Proteobacteria 1013 | Burkholderiales 931 | Comamonadaceae 568 | Acidovorax 125 |  |
|  |  |  | Burkholderiaceae 265 | Cupriavidus 262 |  |
|  | gamma-Proteobacteria  1213 | Xanthomonadales 371 |  |  |  |
|  |  | Enterobacteriales 363 |  |  |  |
| Actinobacteria  2775 | Actinobacteria 2201 | Actinomycetales 2161 | Corynebacteriaceae 740 | Corynebacterium 740 |  |
|  |  |  | Propionibacteriaceae 396 | Propionibacterium 396 | P. acnes 396 |
|  |  |  | Mycobacteriaceae 157 | Mycobacterium 157 |  |
|  |  |  | Micrococcaceae 113 | Kokuria 113 | K. rhizophila 113 |
|  | Acidimicrobia 350 |  |  |  |  |
| Bacteroidetes 894 | Cytophagia 736 | Cytophagales 736 | Cytophagaceae 736 |  |  |
| Firmicutes 652 | Bacilli 552 | Bacillales 183 | Staphylococcaceae 183 | Staphylococcus 183 | S. aureus 183 |
| Acidobacteria 277 |  |  |  |  |  |
| Chloroflexi 165 |  |  |  |  |  |
| Verrucomicrobia 165 |  |  |  |  |  |

minimal OTU count = 10

Table 3S

Total classified reads obtained for specimen 3 assigned to bacterial taxons

| Phylum | Class | Order | Family | Genus | Species |
| --- | --- | --- | --- | --- | --- |
| Proteobacteria 153670 | alpha-Proteobacteria 149370 | Rhizobiales 148092 | Bradyrhizobiaceae 137885 | Bosea 373 | B. genosp. 373 |
|  |  |  | Rhizobiaceae 6665 |  |  |
|  |  |  | Hyphomicrobiaceae 2815 | Devosia 2683 |  |
|  |  | Sphingomonadales 746 | Sphingomonadaceae 0.47 | Sphingopyxis 365 | S. alaskensis 365 |
|  | beta-Proteobacteria 3074 | Burkholderiales 3024 | Comamonadaceae 2797 | Variovorax 1411 | V. paradoxus 1409 |
|  |  |  |  | Acidovorax 685 |  |
|  | gamma-Proteobacteria 1041 | Xanthomonadales 683 |  |  |  |
| Actinobacteria 3017 | Actinobacteria 2416 | Actinomycetales 2416 | Actinosynnemataceae 1048 |  |  |
|  |  |  | Promicromonosporaceae 410 | Promicromonospora 410 |  |
|  |  |  | Propionibacteriaceae 189 | Propionibacterium 189 | P. acnes 189 |
|  | Thermoleophilia 380 |  |  |  |  |
| Bacteroidetes 1141 | Cytophagia 547 | Cytophagales 547 |  |  |  |
| TM7 475 | TM7-1 392 |  |  |  |  |
| Acidobacteria 431 |  |  |  |  |  |
| Chloroflexi 278 |  |  |  |  |  |
| Verrucomicrobia 232 |  |  |  |  |  |

minimal OTU count = 10

Table 4S

Total classified reads obtained for specimen 4 assigned to bacterial taxons

| Phylum | Class | Order | Family | Genus | Species |
| --- | --- | --- | --- | --- | --- |
| Proteobacteria 143731 | alpha-Proteobacteria 120204 | Rhizobiales 117721 | Bradyrhizobiaceae 111684 | Bosea 1548 | B. genosp. 1548 |
|  |  |  | Rhizobiaceae 2090 |  |  |
|  |  | Sphingomonadales 826 | Sphingomonadaceae 826 | Sphingopyxis 826 |  |
|  | gamma-Proteobacteria 16237 | Pseudomonadales 14276 | Pseudomonadaceae 14249 | Pseudomonas 14249 |  |
|  | beta-Proteobacteria 6645 | Burkholderiales 6240 | Comamonadaceae 5827 | Variovorax 2158 | V. paradoxus 2154 |
|  |  |  |  | Acidovorax 595 |  |
|  |  |  |  | Roseateles 339 | R. depolymerans 339 |
| Actinobacteria 7875 | Actinobacteria 4509 | Actinomycetales 4470 | Microbacteriaceae 1771 | Agromyces 1071 |  |
|  | Thermoleophilia 1641 |  |  |  |  |
| Bacteroidetes 4300 | Sphingobacteria 2306 | Sphingobacteriales 2306 | Sphingobacteriaceae 2145 | Pedobacter 2014 |  |
| Acidobacteria 1914 | Acidobacteria-6 1467 | iii1-15 1416 |  |  |  |
| Verrucomicrobia 1094 |  |  |  |  |  |
| Chloroflexi 837 |  |  |  |  |  |
| Planctomycetes 415 |  |  |  |  |  |

minimal OTU count = 10

Table 5S

Total classified reads obtained for specimen 5 assigned to bacterial taxons

| Phylum | Class | Order | Family | Genus | Species |
| --- | --- | --- | --- | --- | --- |
| Proteobacteria 180198 | alpha-Proteobacteria 176087 | Rhizobiales 175360 | Bradyrhizobiaceae 174374 |  |  |
|  | beta-Proteobacteria 1230 | Burkholderiales 888 | Comamonadaceae 604 | Acidovorax 206 |  |
|  |  |  | Burkholderiaceae 249 | Cupriavidus 249 |  |
|  | gamma-Proteobacteria 2282 | Pseudomonadales 1536 | Pseudomonadaceae 1370 | Pseudomonas 1370 |  |
|  | delta-Proteobacteria 599 | Myxococcales 464 |  |  |  |
| Actinobacteria 3471 | Actinobacteria 2407 | Actinomycetales 2366 | Nocardioidaceae 554 |  |  |
|  |  |  | Corynebacteriaceae 447 | Corynebacterium 447 |  |
|  |  |  | Propionibacteriaceae 304 | Propionibacterium 304 | P. acnes 304 |
|  |  |  | Micrococcaceae 146 | Arthrobacter 146 |  |
|  | Thermoleophilia 738 | Gaiellales 475 | Gaiellaceae 475 |  |  |
| Firmicutes 712 | Bacilli 600 | Bacillales 576 | Staphylococcaceae 283 | Staphylococcus 283 | S. aureus 277 |
| Acidobacteria 690 |  |  |  |  |  |
| Bacteroidetes 549 |  |  |  |  |  |
| Chloroflexi 456 |  |  |  |  |  |

minimal OTU count = 10

Table 6S

Total classified reads obtained for specimen 6 assigned to bacterial taxons

| Phylum | Class | Order | Family | Genus | Species |
| --- | --- | --- | --- | --- | --- |
| Proteobacteria 148653 | alpha-Proteobacteria 76462 | Rhizobiales  74991 | Bradyrhizobiaceae  69066 |  |  |
|  |  |  | Rhizobiaceae 4890 |  |  |
|  | gamma-Proteobacteria 52123 | Pseudomonadales  51479 | Pseudomonadaceae  51436 | Pseudomonas  51436 |  |
|  | beta-Proteobacteria  19525 | Burkholderiales 19517 | Comamonadaceae  11840 | Acidovorax  5848 |  |
|  |  |  |  | Variovorax  2242 | V. paradoxus  2242 |
|  |  |  | Oxalobacteraceae  5370 |  |  |
|  |  |  | Burkholderiaceae 79 | Cupriavidus 79 |  |
| Bacteroidetes 17019 | Sphingobacteria 9401 | Sphingobacteriales 9401 | Sphingobacteriaceae  9401 | Pedobacter  9390 |  |
|  | Flavobacteria 5698 | Flavobacteriales 5698 | Flavobacteriaceae 5647 | Flavobacterium 5647 |  |
|  | Cytophagia 1563 | Cytophagales 1563 |  |  |  |
| Actinobacteria 1822 | Actinobacteria 1345 | Actinomycetales 1345 | Micrococcaceae | Arthrobacter 160 |  |
| Firmicutes 185 |  |  |  |  |  |
| Chloroflexi 149 |  |  |  |  |  |
| Acidobacteria 61 |  |  |  |  |  |
| Planctomycetes 60 |  |  |  |  |  |

minimal OTU count = 10
