# Supplementary material for: Identification of bacteria and fungi inhabiting fruiting bodies of Burgundy truffle (Tuber aestivum Vittad.)
Source: Arch Microbiol. 2020 Jul 30;202(10):2727–38. doi: 10.1007/s00203-020-02002-x (PMC7538415; doi:10.1007/s00203-020-02002-x)
Supplement: Supplementary file 2 — Supplementary file2 (DOCX 15 kb) [file 203_2020_2002_MOESM2_ESM.docx]

Table 7S

Number of total classified reads obtained for all specimens assigned to fungal taxa

| Taxonomic classification | | | | | | Specimen no. | | | | | |
| --- | --- | --- | --- | --- | --- | --- | --- | --- | --- | --- | --- |
| Phylum | Class | Order | Family | Genius | Species | 1 | 2 | 3 | 4 | 5 | 6 |
| Ascomycota | Pezizomycetes  Dothideomycetes  Sordariomycetes | Pezizales  Capnodiales  Hypocreales  Melanosporales | Tuberaceae  Mycosphaerallaceae  Hypocreaceae  Ceratostomataceae | Tuber  Mycosphaerella  Trichoderma  Sphaerodes | T. aestivum  M. tassiana  T. neokoningii  S. fimicola | 115701  3  85 | 96745  7  5 | 99867 | 104111  19  51 | 84866  4 | 97593  7 |
| Basidiomycota | Microbotryomycetes  Ustilaginomycotina | Sporidiobolales  Malasseziales | Sporidiobolates fam incertae sedis  Malasseziaceae | Sporobolomyces  Malassezia | S. roseus  M. restricta | 163  3 | 1 |  | 1 | 6 | 3 |
| Mucoromycota | Mucoromycotina | unidentified  Mucorales | Umbelopsidaceae | Umbelopsis | U. isabellina | 29 | 1 |  | 5 |  |  |

minimal OTU count = 10
